# Using machine learning with optical profilometry for GaN wafer screening

**DOI:** 10.1038/s41598-023-29107-9

**Published:** 2023-02-27

**Authors:** James C. Gallagher, Michael A. Mastro, Mona A. Ebrish, Alan G. Jacobs, Brendan P. Gunning, Robert J. Kaplar, Karl D. Hobart, Travis J. Anderson

**Affiliations:** 1 U.S. Naval Research Laboratory, 4555 Overlook Ave SW, Washington, DC 20375 USA; 2 NRC Postdoc Fellow Residing at the U.S. Naval Research Laboratory, Washington, USA; 3 Sandia National Laboratories, MS 1086, PO Box 5800, Albuquerque, NM 87185 USA

**Keywords:** Electronic devices, Electrical and electronic engineering

## Abstract

To improve the manufacturing process of GaN wafers, inexpensive wafer screening techniques are required to both provide feedback to the manufacturing process and prevent fabrication on low quality or defective wafers, thus reducing costs resulting from wasted processing effort. Many of the wafer scale characterization techniques—including optical profilometry—produce difficult to interpret results, while models using classical programming techniques require laborious translation of the human-generated data interpretation methodology. Alternatively, machine learning techniques are effective at producing such models if sufficient data is available. For this research project, we fabricated over 6000 vertical PiN GaN diodes across 10 wafers. Using low resolution wafer scale optical profilometry data taken before fabrication, we successfully trained four different machine learning models. All models predict device pass and fail with 70–75% accuracy, and the wafer yield can be predicted within 15% error on the majority of wafers.

## Introduction

It has been well established in the field of wide bandgap semiconductors that GaN has the potential to surpass Si and SiC based technologies in high power electronic applications^1^. This is due to the high mobility, allowing for higher frequencies and thus smaller components in the electronic circuit^2^, and larger critical electric field allowing for use of shorter distances in field limited applications resulting in a lower on-resistance^3–5^. One major objective in GaN research is to reliably manufacture vertical devices suitable for high power electronics as this would improve the resultant system level size, weight, and power^6^. There are still many challenges to manufacturing PiN GaN wafers needed for many device topologies. Presently, there are many defects in 2 in. GaN commercial wafers^7^, and the homoepitaxial growth of GaN commonly presents carbon defects, threading dislocations, pinholes and hillocks^4,8–10^.

Many non-destructive techniques are useful for detecting defects. Raman spectroscopy is effective at probing changes in conductivity in the substrate by measuring the location of the A_1_ (LO) peak and measuring changes in crystal stress using the E_2_ peak^7,11,12^. Photoluminescence is useful for probing defects at the surface due to the short absorption length of above bandgap light in GaN. X-ray topography is excellent at detecting individual or clustered dislocations, and two photon absorption mapping is good at determining the carrier lifetime, which is related to the defect concentration.

Optical profilometry has many advantages. Most notably, it can scan a 2 inch wafer at micron resolution in a few hours, and does not require an expensive vacuum system for operation. It is effective at detecting defects because many defects manifest themselves as abnormal surface morphology. However, the analysis is not straightforward as several types of defects are benign thus have little effect on device performance^12^. This project studied the defects measured using optical profilometry and correlated them with the performance of vertical PiN diodes.

Machine learning has proven to be useful for making predictions, which are hard to quantify with traditional programming techniques. Due to the large amount of data required, its use in the semiconductor industry is limited. However, research has been gaining much traction recently. There have been many successful results including the prediction of thermoelectric properties^13^, classification using photoluminescence^14^, prediction of AlGaN/GaN HEMT device parameters^15–17^, predicting the quality of GaN Ohmic contacts^18^, and prediction of current–voltage (IV) and capacitance–voltage (CV) data using computationally generated data with TCAD^19–22^. To our knowledge, this is the first report using machine learning on vertical GaN devices trained with experimental data designed for wafer screening.

## Experimental details

### Sample fabrication

**Figure 1:**
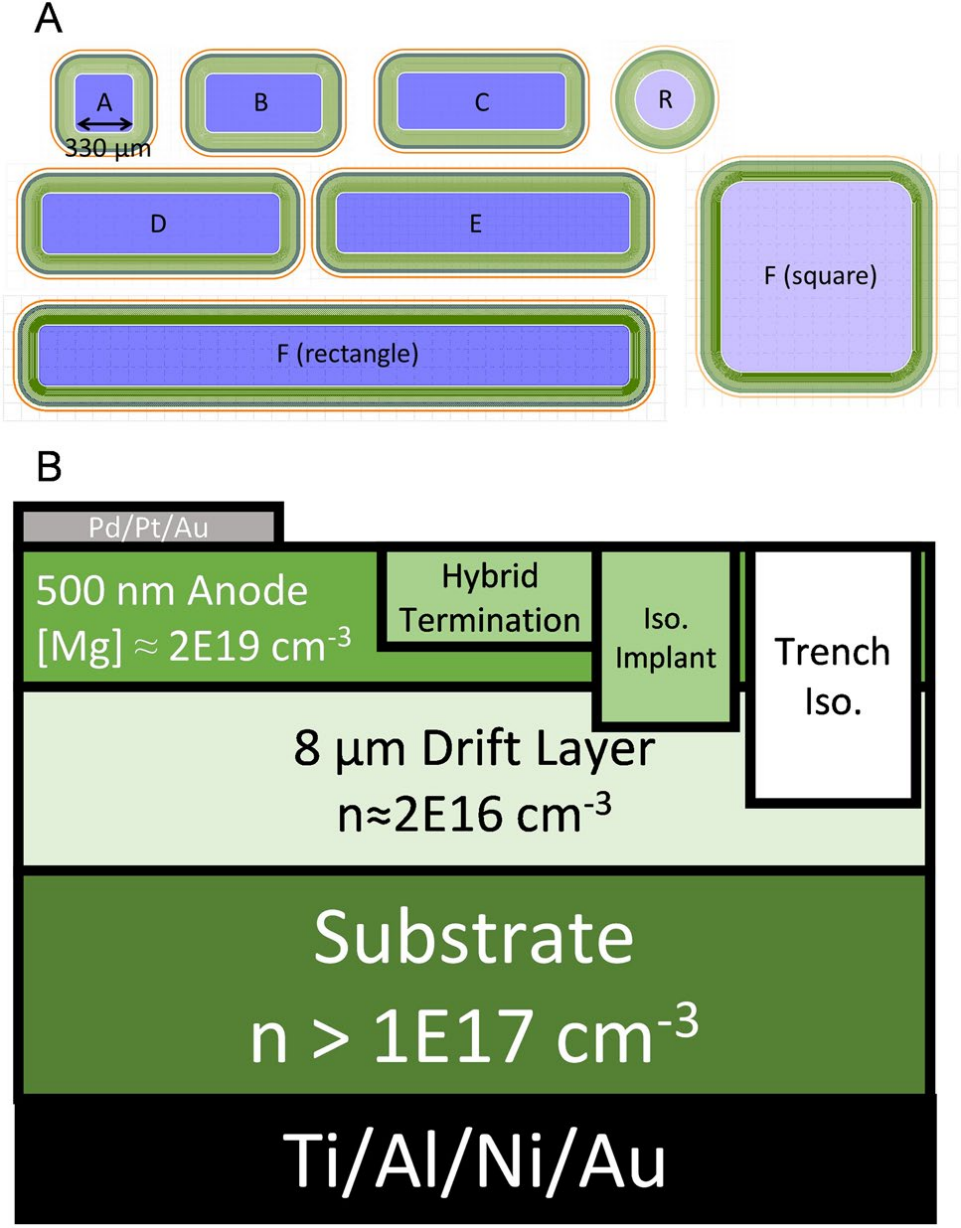
(**A**) To-scale images of the devices used in this study. The blue area represents the anode, the green area represents the guard ring/JTE hybrid termination with an implant isolation layer at the edge, and the orange ring represents the trench isolation region. The areas for the devices are as follows: (A)-0.116 × 10^–2^ cm^−2^, (B)-0.227 × 10^–2^ cm^−2^, (C)-0.338 × 10^–2^ cm^−2^, (D)-0.499 × 10^–2^ cm^−2^, (E)-0.560 × 10^–2^ cm^−2^, (F) (both rectangle and square)-1.11 × 10^–2^ cm^-2^, (R)-0.0911 × 10^–2^ cm^−2^. (**B**) Side view of vertical diode with center at the left (not drawn to scale).

The P-i-N diodes were fabricated by a process described in our previous work^23–25^. Two GaN layers were fabricated in-situ using the Taiyo Nippon Sanso MOCVD SR4000HT reactor at Sandia National Laboratories on ten different GaN substrates using metal organic chemical vapor deposition (MOCVD): an 8 µm drift layer, doped with Si at n ≈ 2 × 10^16^ cm^−3^ and a subsequent p-layer approximately 500 nm thick, doped with [Mg] ≈ 2 × 10^19^ cm^−3^ with an estimated hole concentration p ≈ 5 × 10^16^ cm^−3^ at room temperature constituted the epi stack. Vertical diodes were fabricated with many shapes and sizes. Each wafer had multiple device sizes with an even special distribution across the wafer (see Table 1 for exact number of devices). The exact distribution devices and sizes can be determined from the x–y data in the supplemental materials (“Training Data.csv”). All versions of the diodes had a trench etched outside the devices using an Ar/Cl_2_ plasma for isolation, a ~ 600 nm multi-energy nitrogen implant with a box profile for further isolation within the trench, and an implanted guard ring/JTE hybrid termination^26^ approximately 300 nm deep also created with nitrogen implantation. Ohmic contacts were deposited using Pd/Pt/Au on the p-layer and Ti/Al/Ni/Au on the substrate. The sample device cross section is shown in Fig. 1b.

**Table 1:** This table shows the device types (see Fig. 1a) their area, the number of devices of each time across all 10 wafers, the percentage of devices that passed both forward and reverse criteria of each type, and the accuracy of the model using a two layer neural network model constructed by using halve of the devices of the size of interest as the validation test and the remaining data as the training data set.

Device type	Area (cm^2^)	Number of devices	Percent pass	Model accuracy (%)
R	9.11E−04	153	96.7	96
A	1.16E−03	1777	72.5	77
B	2.27E−03	1476	65.6	75
C	3.38E−03	792	60.7	77
D	4.49E−03	728	58.9	77
E	5.60E−03	724	50.7	71
F	1.11E−02	720	35.5	74

### Data collection

**Figure 2:**
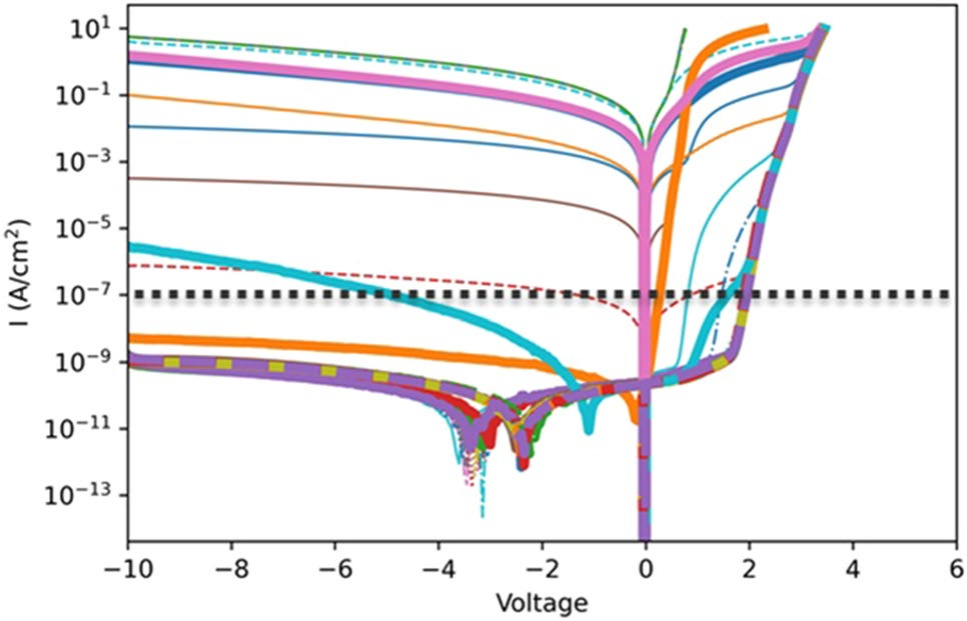
The diode IV curves are taken for all the F (square) devices measured. The dotted line represents the cutoff point for determining if the reverse bias leakage is good at −10 V in this study.

Optical profilometry measurements were taken using Zygo™ NewView 7300 optical profilometer with a 2.5× magnification giving an x–y resolution of 4421 nm/pixel. With each sample, several images were stitched together to map the full 2 inch wafer using Zygo stitching algorithm in their MetroPro software with 25% image overlap to minimize sticing artifacts, which are not noticeable in experimental data. These measurements were taken before any lithography steps were performed. The DC-IV measurements were taken using a Keithley 4200 SMU with a preamplifier, which have a 10 Α measurement resolution. Measurements were taken from −10 V to compliance (at 0.1 A) allowing for the reverse leakage, ideality factor, on-resistance, and turn-on voltage to be calculated for each sample. The performance of the devices varied drastically. Figure 2 shows all the diodes’ IV curves of F-sized (see Fig. 1a) devices on one wafer. Both the forward bias behavior and the reverse leakage current vary drastically.

### Data analysis

For machine learning models to be trained, input (measured variables) and output (test data) variables must be determined. For this project, the optical profilometry data served as the input data and the electrical properties of the diode served as the output data. This section discusses how the data is organized for the machine learning models. The data used to train the models is available in the supplemental materials.

### Analysis of optical profilometry data

**Figure 3:**
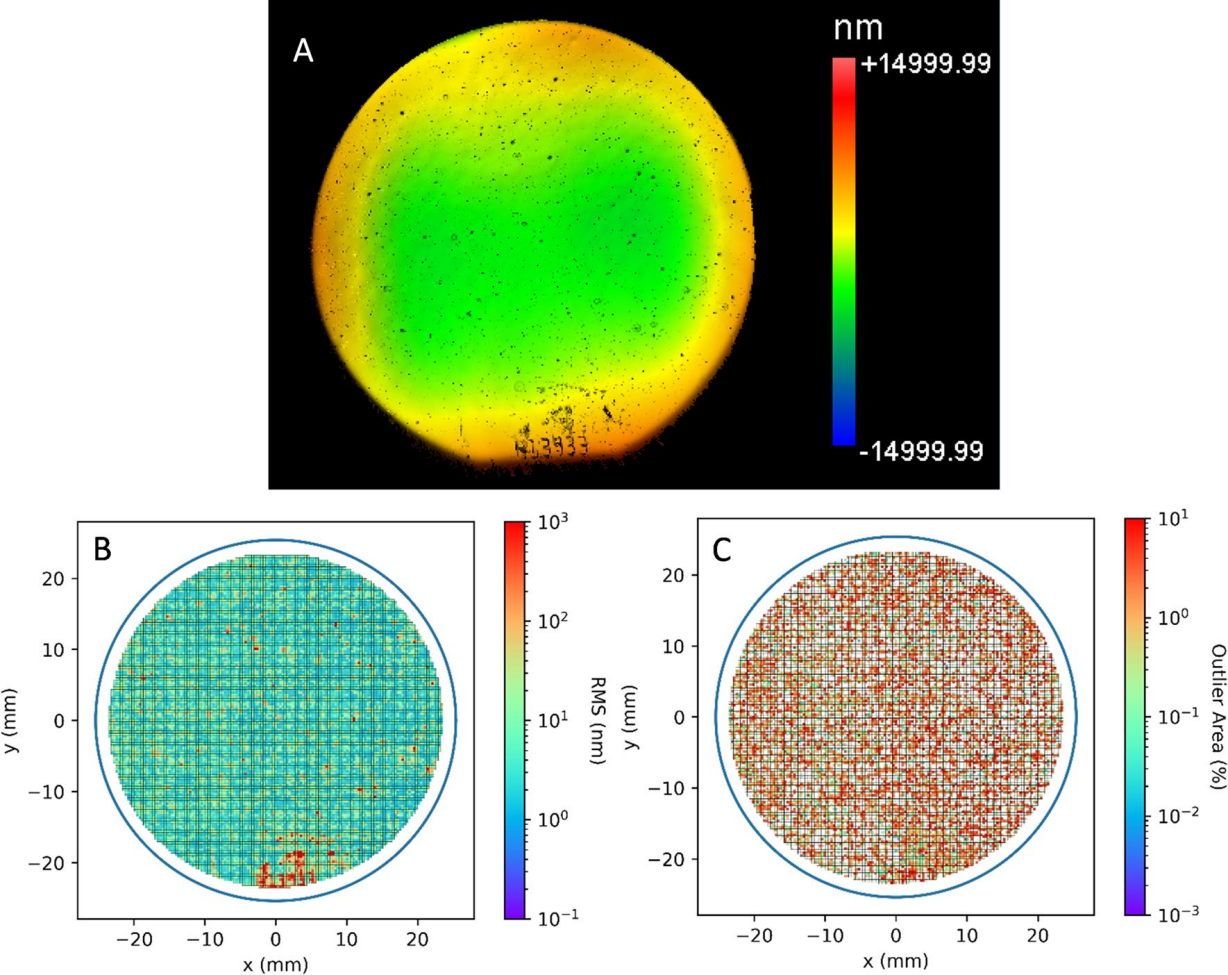
(**A**) Optical Profilometry image of a 2 in. wafer. (**B**) RMS roughness vs position divided into 325 × 325 µm^2^ squares. (**C**) Same as (**B**) except outlier area is in percent of pixels.

**Figure 4:**
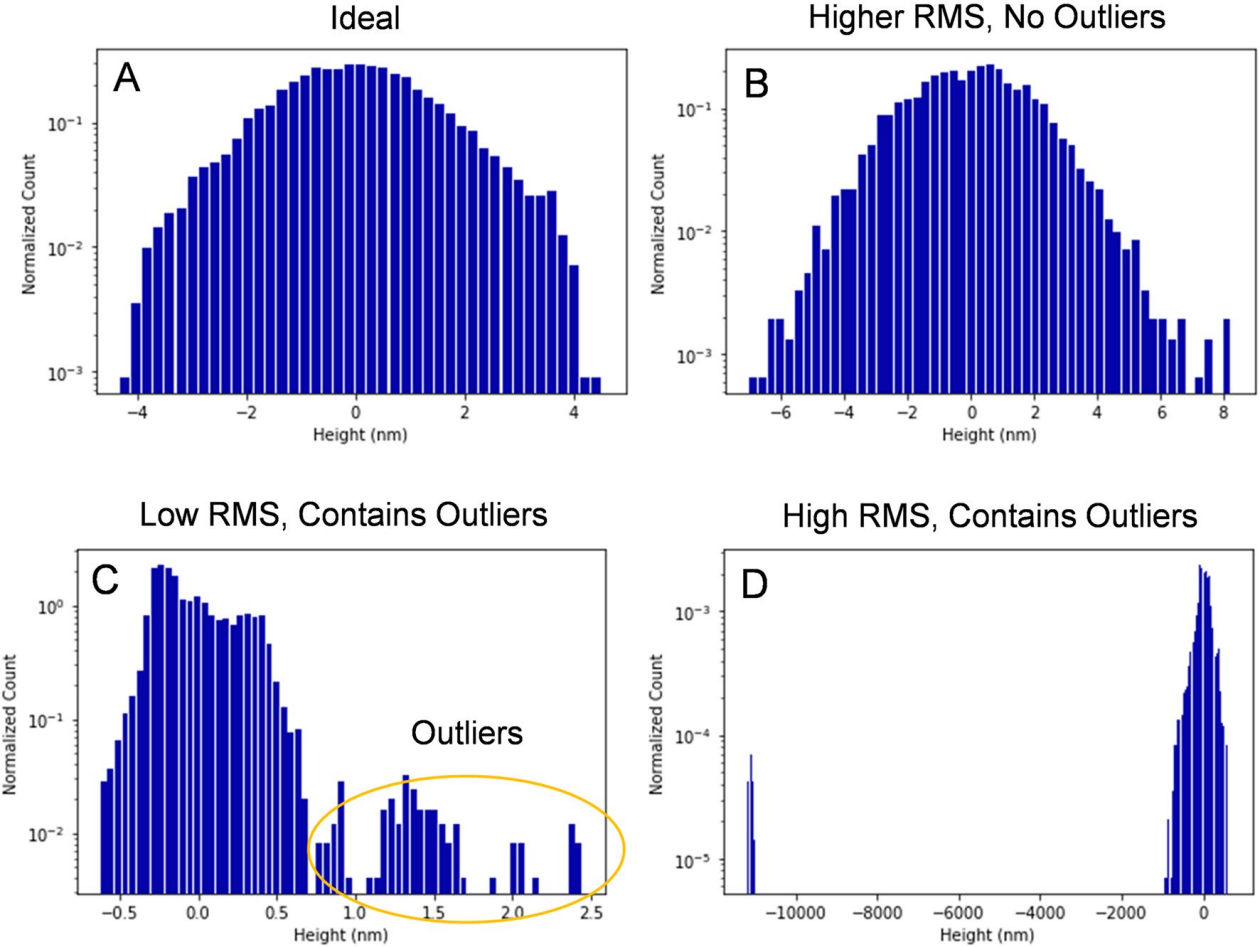
Log scale histogram plot showing the distribution of points in 4 different types of 325 × 325 µm^2^ regions: (**A**) An ideal region with a low RMS value in a Gaussian distribution, (**B**) points are in a Gaussian, but a higher RMS value is measured, (**C**) a region with low RMS, however there are many outliers outside of the Gaussian indicating a small bump typically resembling a red spot in Fig. 3C, (**D**) a region with a very large pit typically resembling an area with a red pixel in (**B,C**).

The optical profilometry (Fig. 3a) data for each wafer was divided into square regions of 325 × 325 µm^2^, which is the size of the anode in A type devices. This region is small enough to allow planar background subtraction. Outlier points were excluded from the background subtraction. In a good region it is expected that z (height) values approximately follow a Gaussian distribution. After the planar subtraction, two numbers were obtained. The first is the root mean square ($RMS=\sqrt{\frac{1}{N}\sum_{i=1}^{N}{\left({Z}_{i}-{Z}_{avg}\right)}^{2}}$), which is a measure of overall roughness of the area. The second is the number of outlier points detected with a generalized Extreme Studentized Deviation (ESD) test^27^ (example in Fig. 3c). These points were used to identify the location of bumps and pits on the sample. According to the space plots of the RMS and outlier area in Fig. 3b,c, these numbers can vary greatly based on the position of the wafer. Figure 4 shows histogram plots of the z-height in 4 different anode-sized squares. A good region (Fig. 4a) has the z-height follow a Gaussian distribution; however, it is not uncommon for the RMS to be larger but still following a Gaussian as shown in Fig. 4b. Sometimes bumps or pits can cause outliers to occur. These also vary in size as demonstrated in Fig. 4c,d. All regions within a 2 mm radius of the sample’s location are averaged in order to account for inaccuracies in the position of the data and account for the effect of defects outside the anode area that affect the performance.

### Analysis of electrical measurements

The machine learning techniques used in this study are classification techniques, thus pass-fail criteria were needed to be set for the DC-IV measurements. Using a DC-IV measurement, a few diode quantities were calculated. First, the leakage current at -10 V was used for the reverse bias assessment (see Table 2). A suitable device required leakage current density below 10^–7^ A/cm^2^, i.e., J ≤ 10^–7^ A/cm^2^ (dotted line in Fig. 2). The ideality factor (η ≤ 2.5), specific on resistance (R_on_ ≤ 50 mΩ-cm^2^), and turn on voltage (2.83 ≤ V_on_ ≤ 3.83) were also used as criteria of passing devices.

**Table 2:** This table shows the RMS error of the yield prediction (see Fig. 8) for four machine learning models.

	Decision tree	Logistic regression	KNN K = 200	2 layer neural network
Reverse bias prediction Error (%)	11.16	16.15	12.36	10.24
Forward bias prediction Error (%)	18.68	15.89	18.11	15.76
Overall prediction error (%)	17.45	14.92	16.28	14.56

### Machine learning models

**Figure 5:**
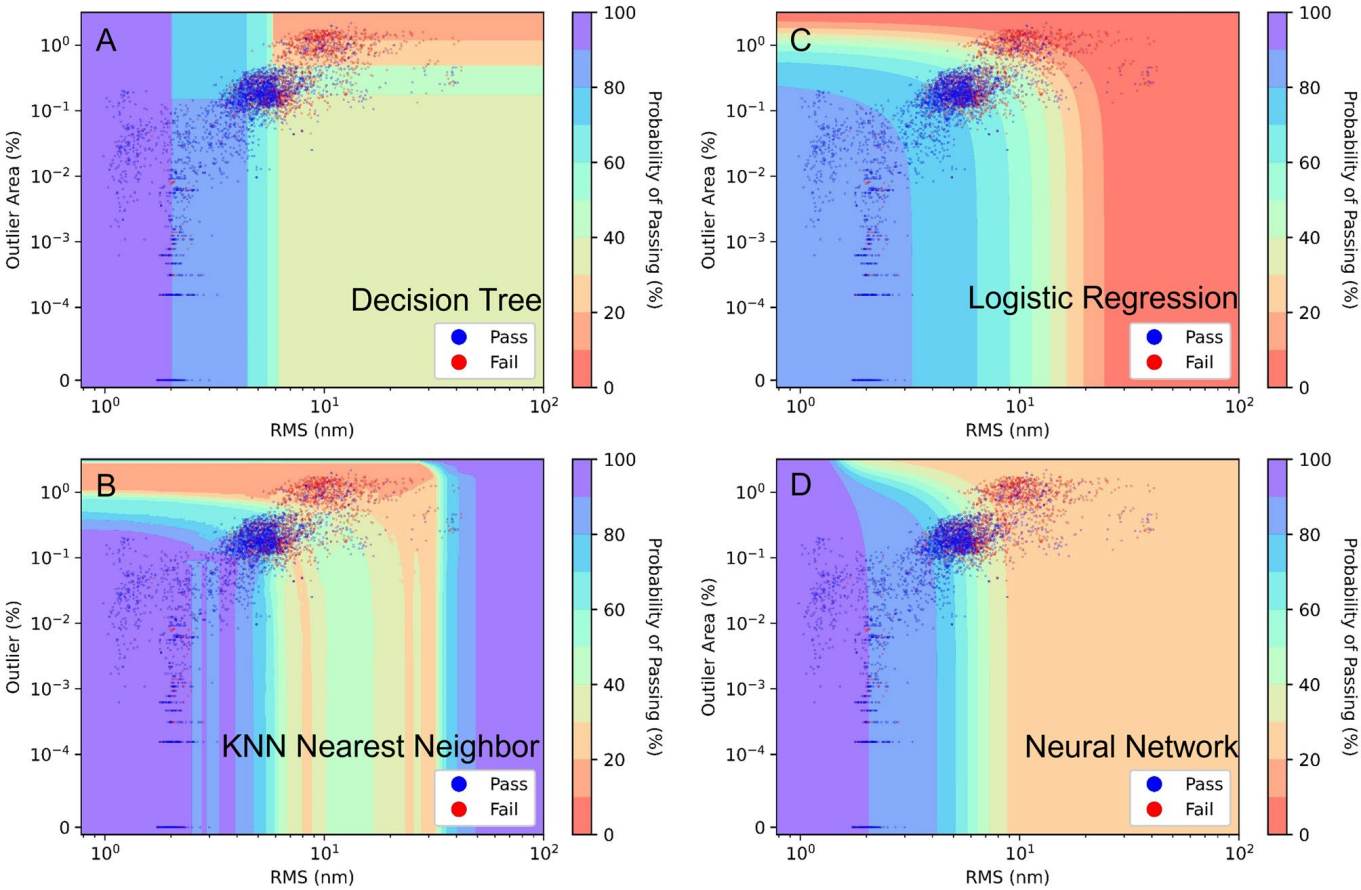
Cluster plots of all devices using the RMS roughness and the number of outliers (Bumps or Pits) on the X and Y axes. Devices in blue pass all (both forward and reverse) criteria, while the red fail any of the criteria. The probabilistic decision boundaries calculated using the four models: (**A**) decision tree, (**B**) KNN nearest neighbor (N = 200), logistic regression (**C**), and (**D**) neural network (2 layer, 3 neurons per layer with logistic activation function). Note that device area and distance from the center are not considered in determining the decision boundaries.

**Figure 6:**
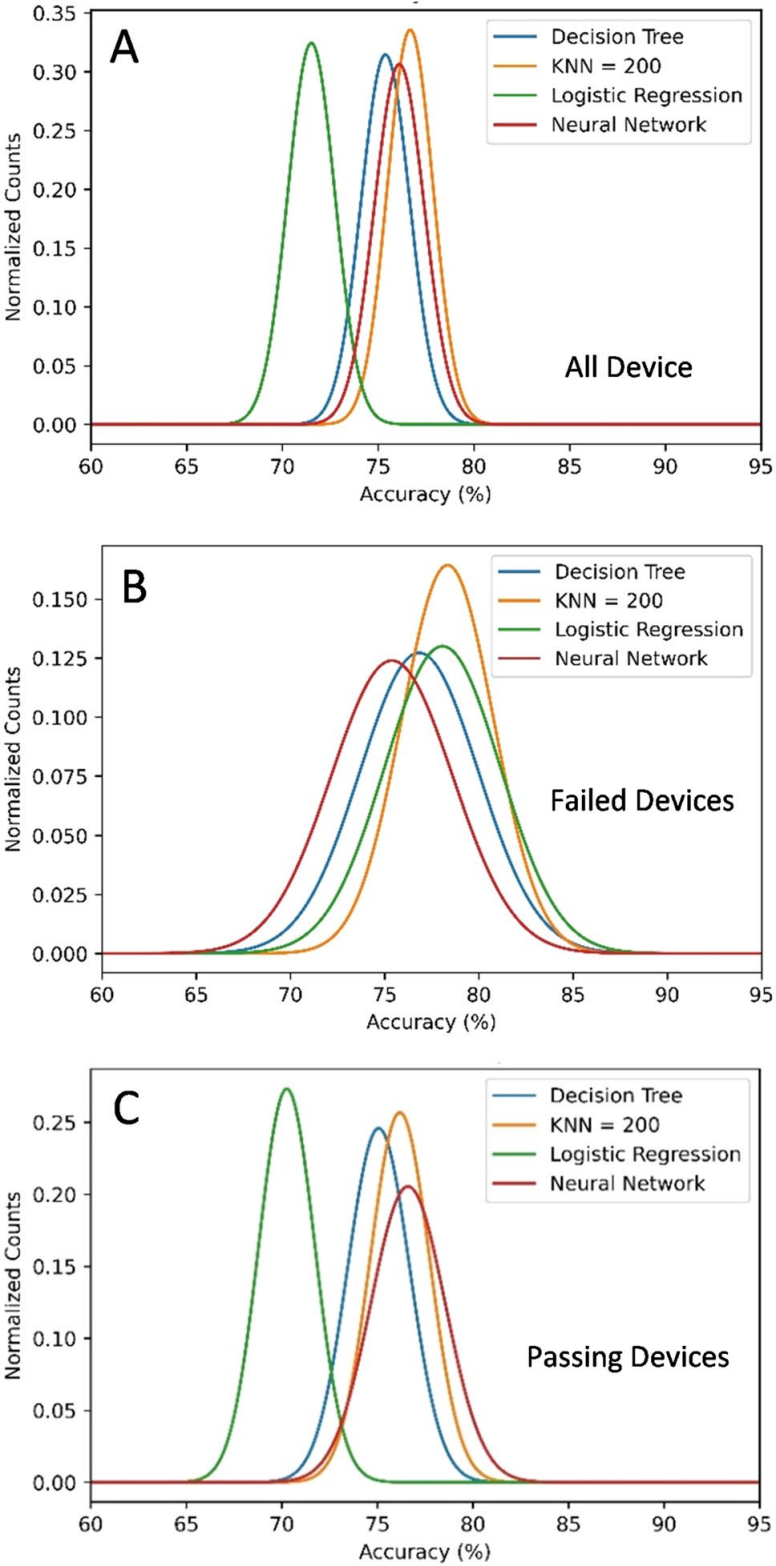
Gaussian fit of accuracy vs number of counts calculated from 1000 training iterations where 80% of the data was used to train the model, and the other 20% was used to test the model chosen at random. (**A**) The accuracy if all data is considered. (**B**) The accuracy of only the devices predicted to fail. (**C**) The accuracy of only the devices predicted to pass.

Four machine learning models were tested in this study. All produced consistent results. These include a (1) Decision Tree, (2) KNN nearest neighbors, (3) Logistic Regression, and (4) a 2 layer neural network^28–31^. All these models were built and tested using the Sklearn package in Python. The models all predict the probability of passing. Figure 5 shows the contour plot of the passing probability overlaid on a cluster plot of RMS vs outlier area. With each model, 80% of the devices were set up to be part of the training data, and 20% were part of the test data selected at random. This test was repeated 1000 times, and Fig. 6a shows the accuracy of the tests fit to a Gaussian. It is important in machine learning to confirm the model is good at predicting all classifications accurately. Therefore, the test was repeated but only considered the failing (Fig. 6b) and passing (Fig. 6c) devices, and similar accuracies were obtained.

**Figure 7:**
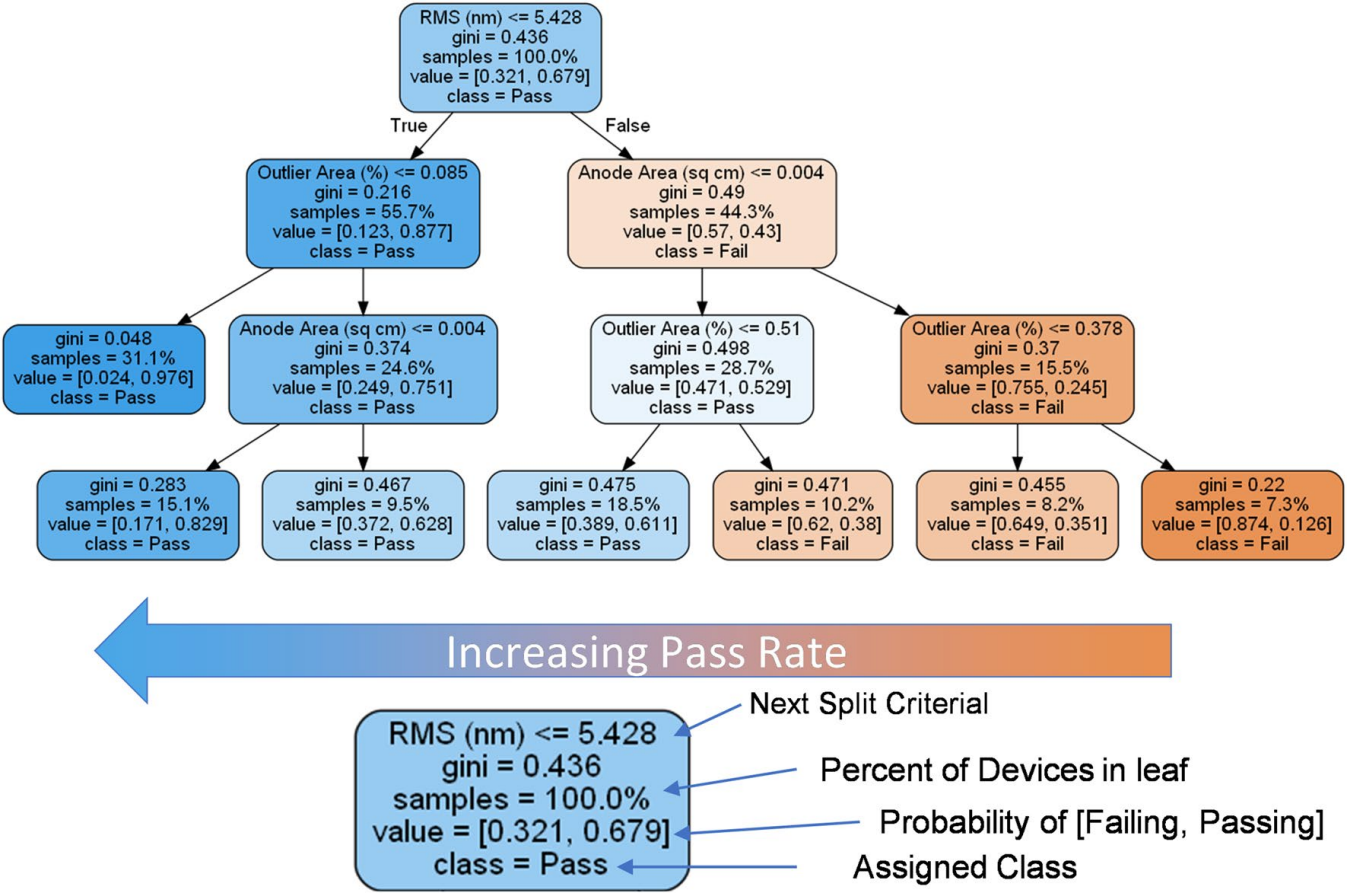
Example decision tree constructed from the Sklearn module in Python using the data. The data are split into leafs which minimize the Gini index. For simplicity this chart is shown to a max depth of 3, though a higher depth of 8 was found to produce the test accuracy. A key showing the information contained in a sample leaf is shown at the bottom.

For the Decision Tree model, the data were split into two leafs across a single variable which minimizes the Gini^32^ index:

$$gini=1-{P}_{pass}^{2}-{P}_{fail}^{2},$$

with ${P}_{pass}$ and ${P}_{fail}$ being the probability of a device in the leaf passing or failing. Splits based on minimizing the entropy index^29^

$$entropy=-{P}_{pass}*lo{g}_{2}\left({P}_{pass}\right)-{P}_{fail}*lo{g}_{2}\left({P}_{fail}\right),$$

produce similar results. The data were split among a single variable at the position which minimizes the weighted average Gini value of the resulting leafs. This technique is useful for identifying which variables are most important for classification. An example decision tree is shown in Fig. 7. The tree shows that the RMS roughness is the most influential variable, followed by outlier area and device area which are of about equal importance. The distance from the center of the wafer appears to be an irrelevant variable likely because not many devices were fabricated close to the edge, thus this variable was removed from analysis for the other methods. Figure 5a shows the probabilistic decision boundary of a decision tree model. In general, this model produces a higher probability of passing with a lower RMS and few bumps and pits. However, there is an exception at high RMS with few bumps and pits. This is likely because high RMS values without bumps or pits present indicates that several defects are present in the sample and would not be triggered as an outlier using the outlier tests.

The K Nearest Neighbor algorithm^30^ was also tested. This method assigns a class (pass/fail) based on the class of the K nearest neighbors (K = 200 was selected for this study) as determined by the distance function $d=\sqrt{\sum_{i}(v{}_{i}-{v}_{test}{)}^{2}}$, where $v{}_{test}$ is the input variable (rms, outlier area, or anode area) of the test point and ${v}_{i}$ are the input variables of the K nearest neighbors. Note that the variables are normalized since they often vary by orders of magnitude. The probability of passing is equal to the percentage of the 200 nearest neighbors which passed. The accuracy test shown in Fig. 6 reveals that this method is the most accurate and is particularly good at predicting failures given by its near 80% average accuracy displayed in Fig. 6b; however, the probability cluster plot in Fig. 5b reveals that this method predicts a high pass rate at extremely high RMS values. This is because this method can have issues with extrapolating results.

Logistic regression^31^ is a one layer neural network which uses the logistic function,

$${P}_{pass}=\frac{1}{1+{e}^{-(\overrightarrow{{w}_{o}+\overrightarrow{ w}\cdot \overrightarrow{v})}}},$$

where $\overrightarrow{v}$ is a vector containing the input variables and the values of vector $\overrightarrow{w}$ , and the constant terms ${w}_{o}$ are the coefficients which are determined by minimizing the mean squared error of the function (L2 normalization). The cluster plot in Fig. 5c shows that this method draws a very logical probabilistic function similar to the one a human expert would draw; however, this method has the lowest accuracy (see Fig. 6). This is likely due to it being a linear model producing a planar probabilistic decision boundary.

The final method involved training a two-layer neural network with three neurons per layer with a logistic activation function in both layers. This method had the 2nd highest accuracy of the four and did not have issue with extrapolated values (see Fig. 5d); however, since it depends on several fit parameters, it has the largest distribution of error as indicated by the full-width half-max of the Gaussian fit in Fig. 6. It is also more difficult to understand what parameters are most important to this model due to the complexity of neural networks^28^. The accuracy of this model was tested as a function of device size by taking half of the devices of each size as test data, and the rest of the data set as training data. The results are shown in Table 1, which showed similar accuracy when predicting all device sizes except for the R sized devices, though this is likely due to the small number of devices with this size.

### Predicting wafer yield

The purpose of this research is to predict the yield on future wafers not included in the training model. The accuracy of the yield prediction was calculated by first training the four models on all the wafers exept the test wafer, then testing the model on the test wafer, and then comparing the experimental yield to the predicted yield:

$${Y}_{pred}=\frac{1}{N}\sum_{i}{{P}_{pass}}_{i}$$

**Figure 8:**
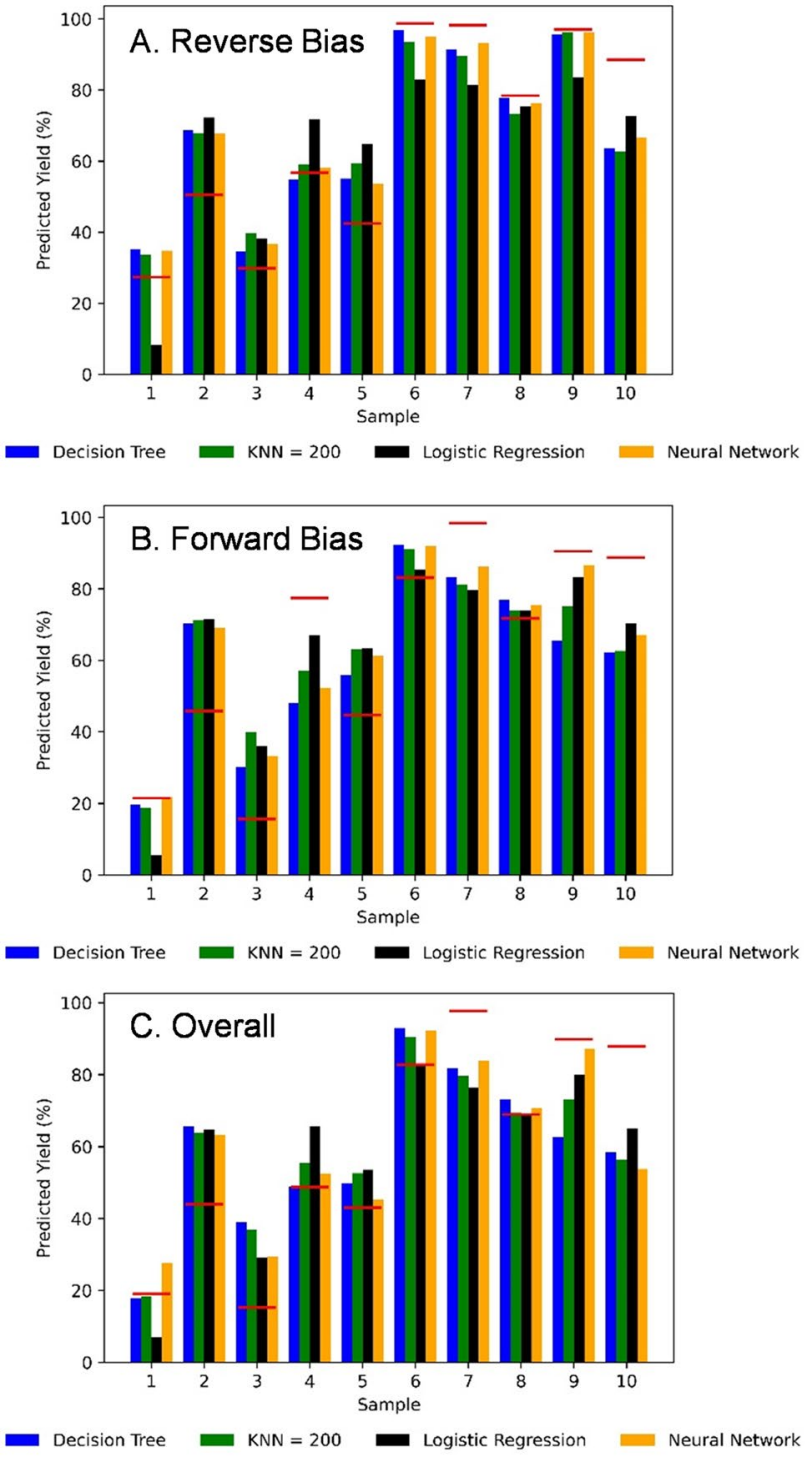
The predicted yield of the four models compared to the experimental results (red line) for predicting the percentage of devices passing the reverse bias criteria (**A**), forward bias criteria (**B**), and both criteria (**C**). The RMS error is shown in Table 2. For each wafer (or sample), the machine learning models were trained using data from the other nine wafers with the wafer being tested to avoid falsely increasing the accuracy by overfitting.

The predicted yield was calculated by taking the average probability that a device passes on the wafer. The results (shown in Fig. 8) reveal that it predicts the error within 15% on 80% of the wafers. The error (see Table 2) was calculated using the Root Mean Square Deviation method:

$$RMSD=\sum_{i}\sqrt{\frac{{\left({Y}_{{exp}_{i}}-{Y}_{pre{d}_{i}}\right)}^{2}}{N}},$$

where ${Y}_{{exp}_{i}}$ is the experimental yield, ${Y}_{pre{d}_{i}}$ is the yield predicted by the model, and N is the number of wafers.

## Discussion

The models all predict that devices are more likely to pass with lower RMS values, a lower number of outliers (bumps and pits) in the area, and a small device size as larger device sizes are more likely to be on a problematic defect. All four models have similar accuracies according to the results in Fig. 6. The most accurate is the KNN model, which is close to 80% accurate. However, the probabilistic decision boundaries are the most abnormal. In particular, the model, shown in Fig. 5b, predicts a success rate at very high roughness values (> 13 nm), while a high fail-rate is expected. This arose because this model can have issues when extrapolating. Logistic regression produces the most logically consistent model, however its accuracy is the lowest likely because logistic regression is restricted to linear probabilistic decision boundaries. The neural network seems to be the best compromise between the two, however it is difficult to assign attribution to the features that the model is considering and has the broadest range of error as indicated by the high full-width half-max of the Gaussian error fit in Fig. 6. The Decision Tree reveals the most about which variables are useful, but can only produce vertical and horizontal probabilistic decision boundaries, which can yield a high prediction yield error as seen in Table 2.

The models are not perfectly accurate for several reasons. First, the models were trained using low resolution (4.42 µm/pixel) optical profilometry data. Many defects are too small to be detected on such a large scale such as point defects. Second, this model does not consider potential errors occurring during processing, only with the starting materials. Third, the experiment contained thousands of samples, which is enough to see if there is a trend, but typically millions are required to train a model suitable for commercial manufacturing. Fourth, our previous research^33^ has shown that many benign defects are present as shown by the cluster plots in Fig. 5 from the large number of points at approximately 0.1% outlier area and an RMS roughness of approximately 5 nm. All models predict close to 50% pass rate in this area. This is likely due to several of the bumps and pits being benign. When predicting the wafer yield, all models predict the reverse bias (Fig. 8a) passing conditions better than forward bias (Fig. 8b) conditions. One possible reason is that reverse bias failure criteria are more easily detected by optical profilometry, as features causing shorts or high leakage are often visible as large bumps or pits in the diode, whereas point defects, not detectableby optical profilometry, may result in changes in carrier concentration resulting in forward bias criterion failure.

This research trained models on the optical profilimetry data for the purpose of wafer screening before the manufacturing of vertical diodes on homoepitaxial GaN. However, it is likely that the same algorithm could be used to train machine learning models for other semiconductor materials and devices as well. First, a (or a combination of several) non-destructive full wafer techniques could be used to map the locations of critical device defects would need to be found then pass-fail criteria would need to be established for the fabricated devices. The defect density variables would be the input data and the pass-fail criteria would be the output data needed to train the model. If the model has a reasonable accuracy, then the defect density variables are accurate and the full wafer mapping technique is useful. Otherwise, a more accurate non-destructive full wafer mapping technique will need to be utilized.

## Conclusion

By fabricating over 6000 vertical GaN diodes on 10 wafers, four different machine learning models were successfully trained using low resolution optical profilometry data. All models were over 70% accurate when predicting whether a device would pass or fail. The models predict true positives with close to the same accuracy as true negatives. When predicting the wafer yield, the model has an RMSD error of ± 15%, and thus is effective at wafer screening.

Despite the limitation and imperfect accuracy, this paper demonstrates a good first step to using machine learning to predict the quality of GaN devices The accuracy of the model could likely be improved by adding other wafer scale nondestructive techniques which are sensitive to changes in doping levels and point defects (such as photoluminescence imaging, Raman spectroscopy, or x-ray topography) or training a different model with an image recognition convolutional neural network to distinguish between benign and problematic defects. In addition, future follow up studies should including testing other diodes properties such as breakdown voltage, junction or capacitance charge, and electrical stress testing and test the effectiveness at predicting other types of devices such as vertical transistors.

## Supplementary Information

Supplementary Information.

## Data Availability

The data to used to train the machine learning models is available in the supplementary materials file “Training Data.csv.” The authors affirm the information needed to reproduce this work is avalible in the published article.
